# The Limitations of Current T Cell-Driven Anticancer Immunotherapies Can Be Overcome with an Original Extracellular-Vesicle-Based Vaccine Strategy

**DOI:** 10.3390/vaccines11121847

**Published:** 2023-12-13

**Authors:** Maurizio Federico

**Affiliations:** National Center for Global Health, Istituto Superiore di Sanità, 00161 Rome, Italy; maurizio.federico@iss.it

**Keywords:** anticancer immunotherapy, extracellular vesicles, immune checkpoint inhibitors, CAR-T cells, anticancer mRNA vaccines

## Abstract

The emergence of tumors associated with defects in immune surveillance often involve the impairment of key functions of T lymphocytes. Therefore, several anticancer immunotherapies have focused on the induction/strengthening of the tumor-specific activity of T cells. In particular, strategies based on immune checkpoint inhibitors, CAR-T cells, and mRNA vaccines share a common goal of inducing/recovering an effective antitumor cytotoxic activity, often resulting in either exhausted or absent in patients’ lymphocytes. In many instances, these approaches have been met with success, becoming part of current clinic protocols. However, the most practiced strategies sometimes also pay significant tolls in terms of adverse events, a lack of target specificity, tumor escape, and unsustainable costs. Hence, new antitumor immunotherapies facing at least some of these issues need to be explored. In this perspective article, the characteristics of a novel CD8^+^ T cell-specific anticancer vaccine strategy based on in vivo-engineered extracellular vesicles are described. How this approach can be exploited to overcome at least some of the limitations of current antitumor immunotherapies is also discussed.

## 1. Introduction

Anticancer immunotherapy interventions help the immune system recover the ability to recognize and destroy tumor cells. Cancer cells can express antigens not produced by healthy cells, i.e., tumor-specific antigens (TSAs) like Human Papilloma Virus-E6 and -E7 in cervical tumors and alpha-fetoprotein in both germ cell tumors and hepatocellular carcinoma. Many TSAs can also be coded well by non-canonical mRNAs arising from epigenetic changes and splicing aberrations. These cancer-specific products, defined as neo-antigens [1], are the molecular targets of most advanced anticancer vaccine strategies. Cancer cells can also upregulate antigens expressed by normal cells (i.e., tumor-associated antigens, TAAs), as in the case of HER2/neu, EGFR, and melanoma-associated antigens.

Both the reactivation and ex novo generation of T cell-driven antitumor activity are the ultimate goals of several anticancer immunotherapies, including those based on immune checkpoint inhibitors (ICIs) [2], chimeric antigen receptor (CAR) T cells [3], and mRNA-based vaccines [4]. At present, ICIs and CAR-T cells are in use in clinics. However, their success is coupled with some still-unresolved issues that need to be overcome. Among these, the most relevant are adverse events, a lack of specificity, tumor escape, and huge costs. These shortcomings call for new solutions able to render anticancer immunotherapy approaches more safe, effective, and affordable.

In this perspective article, the major limitations of current anticancer immunotherapies are detailed together with a means of overcoming at least some of them through the exploitation of a novel, extracellular vesicle (EV)-based, CD8^+^ T cell-specific antitumor vaccine platform. The promising results achieved in pre-clinical experiments using such an immunotherapeutic approach pave a path to its clinical experimentation, the results of which might result in relevant advancements in the fight against cancer.

## 2. Current Immunotherapies Designed to Increase the Anticancer T Cell Activity: ICIs, CAR-T Cells, and mRNA-Based Vaccines

**Figure 1:**
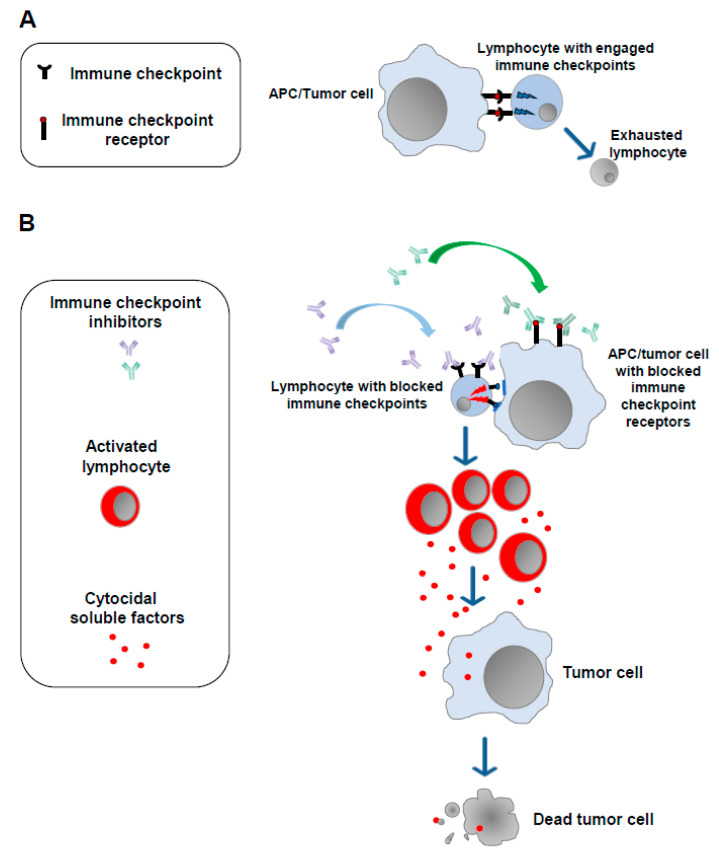
Immune checkpoint inhibitors. (**A**) In the presence of a continuous antigenic stimulus, tumor-specific T lymphocytes upregulate a series of cell membrane molecules (i.e., immune checkpoints), the binding of which with the counter receptors expressed by APCs and tumor cells leads to lymphocyte exhaustion. (**B**) Blocking the immune checkpoints and/or their counter receptors on the cell membrane of APCs and/or tumor cells with monoclonal antibodies (immune checkpoint inhibitors) allows lymphocytes to regain their ability to target and kill tumor cells.

The notion that a strong correlation exists between the emergence of tumors and alterations in the functions of the immune system is now universally accepted [5]. In 2011, results from studies on the mechanisms underlying the modulation of the cell immune response against non-self-antigens were exploited to develop the first anti-tumor immunotherapy based on the administration of antibodies blocking molecules dampening the cell immune response (immune checkpoints). The basis of the use of ICIs is the idea that the persistence of the immunogenic stimulus induced by tumor antigens can lead to a state of exhaustion in T lymphocytes. This exhaustion is marked by the overexpression of a plethora of immune checkpoints whose engagement leads to the loss of antitumor immune surveillance. Blocking such inhibitors using ICIs helps T lymphocytes regain their reactivity against tumor cells (Figure 1).

The first ICIs introduced in clinical practice were antibodies against the immune checkpoint CTLA-4 [6], a transmembrane molecule expressed by lymphocytes which binds both CD80 and CD86 molecules on antigen-presenting cells. It was followed by the successful use in humans of antibodies blocking PD-1, an additional immune checkpoint expressed by exhausted lymphocytes, as well as of its ligands PDL-1 and PDL-2, which are expressed by both antigen-presenting cells (APCs) and tumor cells [7,8,9].

**Figure 2:**
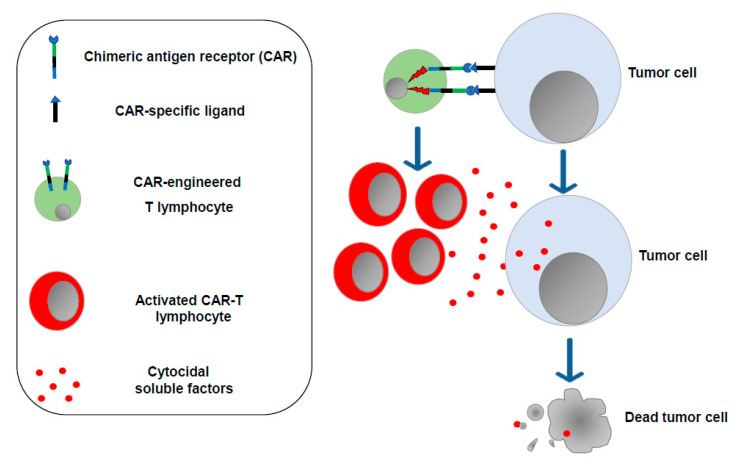
CAR-T lymphocytes. T lymphocytes are isolated from the cancer patient (or from a compatible donor) and transduced in vitro to express the chimeric antigen receptor of choice. After expansion, engineered T lymphocytes are reinfused into the patient, where they can recognize and destroy the tumor cells.

The concept that enforcing the antitumor functions of immune cells would be instrumental in controlling the cancer cell growth was also on the basis of CAR-T cell-based therapy, which consists of the ex vivo generation of T lymphocytes (and, more recently, NK cells as well) [10] expressing a synthetic transmembrane receptor able to bind, mostly through single-chain antibody sequences, protein markers typically expressed on the surface of tumor cells. Upon binding with target cells, the CAR transmits intracellular signals, ultimately leading to lymphocyte activation and proliferation with the release of cytotoxic factors killing target tumor cells (Figure 2).

The first CARs were built as products of the fusion of anti-CD19 single-chain antibodies with the intracellular domains of either CD28 or the TCR-zeta chain [11]. Since then, new generations of CARs have been created, with the newest ones including intracellular signaling sequences from either 4-1BB or CD28 fused with those from the TCR-zeta chain. Typically, the CAR sequences are introduced into T lymphocytes by means of either retro- or lentiviral vector-driven in vitro transduction. Cell sources can be either the patient (autologous cells) or a compatible donor. After expansion, transduced cells are then re-infused in such a way that they become able to recognize tumor cells, most commonly from hematologic neoplasms. In 2017, successful clinical trials led the FDA to approve two CAR-T cell-based immunotherapies for the treatment of B-cell acute lymphoblastic leukemia (B-ALL) and of the diffuse large B-cell lymphoma (DLBCL). At present, six CAR-T cell-based therapies have been approved by the FDA [12] in which the molecular targets of CAR engineered T-cells are CD19 and BCMA (B cell maturation antigen). Afterwards, CD20, CD33, CD123, and FLT-3 were considered for new CAR-T cell-based clinical protocols.

**Figure 3:**
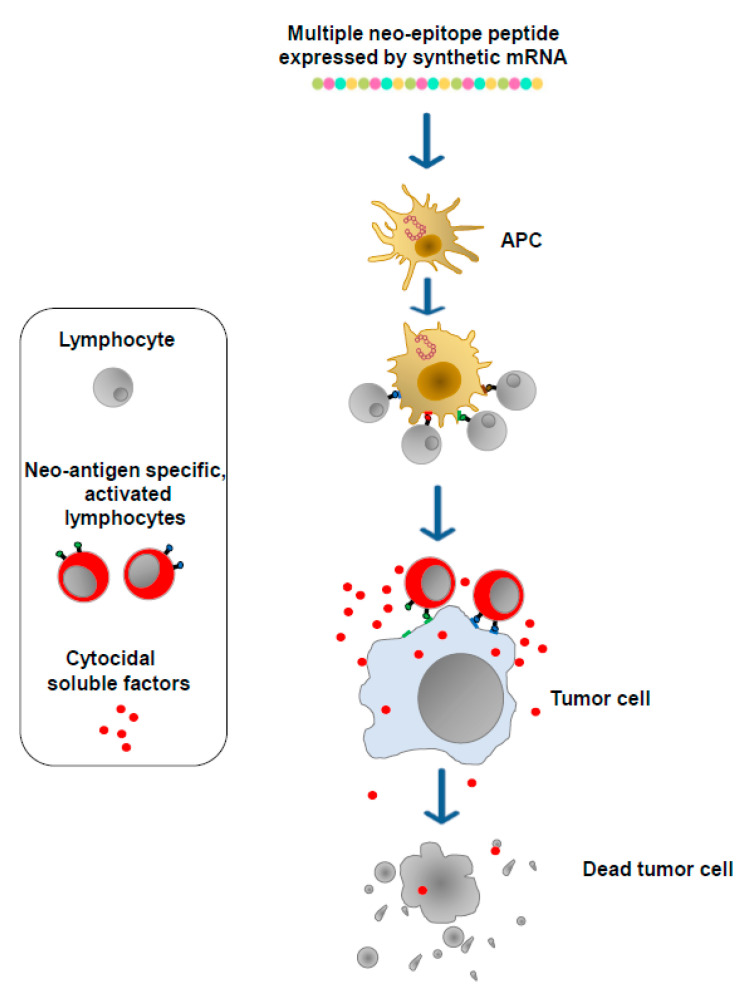
mRNA-based vaccines. Neo-antigens are identified via DNA/RNA sequencing. Dedicated algorithms select neo-antigen amino acid sequences most likely associated with the MHC Class I and Class II molecules of the patient. Sequences coding neo-antigen epitopes are then included in synthetic mRNA molecules (multiple neo-epitope mRNA) which, upon incorporation into lipid nanoparticles, are injected either intradermally, intramuscularly, or intravenously. The neo-epitopes are finally exposed on the MHCs of APCs to induce neo-antigen-specific T lymphocytes.

Cancer cells express, in addition to well-known TSAs and TAAs, a plethora of unidentified proteins defined neo-antigens which bear potentially immunogenic epitopes and whose patterns vary among both patients and tumor types. These products are the targets of mRNA-based antitumor vaccines, which are designed to generate neo-antigen-specific T lymphocytes. To this end, DNA from both the tumor and normal tissues of a patient is sequenced to detect genetic changes unique to the patient’s tumor. Then, to identify neo-antigens, mRNA purified from tumor cells is sequenced to assess which of the observed genetic changes is expressed. Through specific algorithms, candidate neo-antigens, including genetic changes unique to the tumor and potentially associating with either the major histocompatibility complex (MHC) Class I or Class II of the patient, are selected, and the corresponding sequences are included in synthetic mRNA molecules. After association with synthetic lipidic nanoparticles, the mRNA molecules are then injected either intradermally, intramuscularly, or intravenously. In this way, the vaccine nanovesicles can enter any kind of cell. In the case of their entry into APCs, multiple neo-epitope peptides can be processed and presented on MHC complexes soon after translation. Otherwise, multiple neo-epitope peptides can be internalized and processed by APCs after their secretion from target cells. Neo-epitopes presented by dendritic cells (DCs) as well as other APCs select and activate both CD4^+^ and CD8^+^ T lymphocytes which, in this manner, acquire the ability to attack and destroy tumor cells expressing the neo-antigens (Figure 3).

The results from a study carried out in 2017 using such a personalized strategy on melanoma patients produced promising but also unexpected results, considering that the majority of neo-epitopes selected for the CD8^+^ T cell response conversely mounted a CD4^+^ T cell activation [13]. More recently, in a randomized Phase 2b trial, an antitumor combination immunotherapy based on the administration of both an anti-PD-1 IBI and an mRNA-based vaccine demonstrated efficacy against resected III/IV stage melanoma stronger than what was observed after treatment with the IBI alone (Moderna and Merck, accessed 23 February 2023, https://bit.ly/3xJZLiS). Furthermore, data from a phase I clinical trial carried out with an individualized neo-antigen vaccine for pancreatic ductal adenocarcinoma tumors were recently published [14]. In detail, the neo-antigen vaccine was administrated after anti-PD-L1 IBI treatment and before a four-drug chemotherapy regimen. The neo-antigen vaccine was well tolerated and induced de novo neo-antigen-specific T cells in 8 out of 16 patients, with half targeting more than one vaccine neo-antigen.

## 3. Limitations of Current T Cell-Specific Antitumor Immunotherapies

ICI-based immunotherapy is now largely in use in clinical practice, but in many instances, it can induce relevant side effects mostly due to a generalized and poorly controlled activation of the immune cell system. This effect can lead to both acute and chronic autoimmune diseases which can negatively influence the quality of life of the patient [15].

CAR-T cell-based immunotherapy shows relevant limitations in terms of side effects as well, in particular regarding cytokine release syndrome (CRS), and immune effector cell-–associated neurotoxicity syndrome (ICANS) [16,17,18]. CRS consists of an altered/increased release of inflammatory cytokines inducing fever, myalgia, tachycardia, and organ dysfunction, whereas the mechanisms underlying ICANS have not been clarified yet. It is a common and typical toxicity which can accompany and correlate with CRS, but it can also occur independently. Early manifestations of ICANS include expressive aphasia, tremor, dysgraphia, and lethargy. These symptoms can progress to seizures, obtundation, stupor, and coma, possibly evolving to fatal intracerebral hemorrhage and malignant cerebral edema. Both high costs and the selection of null mutant tumor cells (“escape”) represent additional restraints for the CAR-T cell approach.

The limitations of mRNA-based personalized antitumor vaccines include at least two aspects, i.e., the extraordinarily high costs of the procedures and the optimization of the selection of MHC Class I-specific neo-epitopes required to generate an adequate bulk of antitumor cytotoxic CD8^+^ T lymphocytes (CTLs).

## 4. The Extracellular Vesicles

EVs are part of the mechanisms of both short- and long-distance intercellular communication [19]. Different EV subtypes can be distinguished by size and density, sharing, however, a common structure formed by a lipid bilayer membrane including a specific cargo of molecules. According to their biogenesis, two types of EVs can be distinguished, i.e., exosomes and ectosomes/microvesicles. Exosomes are vesicles 30–150 nm in diameter and accumulate in intraluminal vesicles (ILVs) as the result of the inward budding of the endosomal membrane and the generation of the multivesicular bodies (MVBs). Microvesicles are 150–1000 nm in diameter and bud directly from the plasma membrane.

MVB generation is driven by at least two distinct pathways and involves the sorting of various molecules. One pathway involves the endosomal sorting complex required for transport (ESCRT) which is composed of different subunits, namely ESCRT-0 to -III. This molecular complex is recruited to the endosomal membrane, where the individual steps of ILV biogenesis progress. The second pathway of MVB formation involves raft-based lipid microdomains. These are present on the limiting membranes of endosomal compartments and contain high amounts of sphingolipids, which are substrates for neutral sphingomylinase2 [20]. This enzyme modifies sphingolipids into ceramide which, in turn, induces the coalescence of microdomains into more complex structures, thereby promoting the domain-induced budding and formation of ILVs. Then, MVBs can be forwarded to either degradative or secretory pathways. In the latter case, the release of ILVs occurs upon MVB fusion with the plasma membrane.

Conversely, the mechanisms leading to the generation of microvesicles occurring at the plasma membrane are largely unknown. Before shedding, cytoplasmic protrusions undergo fission events, and microvesicles pinch off the cellular membrane. Microdomain-induced budding processes are likely involved in secretion events [21].

Body fluids like blood, amniotic fluid, urine, bronchoalveolar lavage fluid, synovial fluid, breast milk, and saliva contain different types of nanovesicles with distinguished biophysical features and functions in health and disease, e.g., protein clearance, immune regulation, and cell signaling. Therefore, EVs are unique diagnostic biomarkers due to their ability to alter their cargo according to different cell stimuli. In cancer, they can be useful for monitoring disease progression as well as evaluating therapy responses. For example, in melanoma patients, the proteomes of circulating exosomes can be correlated with different clinical tumor stages. In a similar way, a distinctive set of micro-RNA (miRs) uploaded in exosomes marks the evolution of both ovarian and colorectal cancers [22].

In addition, the ability of EVs to overcome natural barriers and their stability in circulation make them effective drug delivery vehicles. The earliest studies carried out using EVs as therapeutics were based on their capacity to modulate immune responses with the intent to develop cell-free cancer vaccines. For instance, DC exosomes carrying melanoma-associated antigen (MAGE)-A3 peptides were used for the vaccination of patients bearing MAGE-A3^+^ advanced melanomas [23].

The EV membrane can be engineered with heterologous proteins in multiple ways. Notably, EVs from ovalbumin-pulsed, activated DCs were modified with an anti-CTLA-4 antibody (i.e., an ICI) to generate bifunctional EVs combining immunization and ICI function [24]. Functionalized EVs could synergize cancer vaccine efficacy and immune checkpoint inhibition to generate potent antitumor immune responses against a tumor.

EVs can be also engineered to incorporate mRNAs and small interfering RNAs (siRNAs) and were proven to be active in strategies of RNAi-based therapies as, for instance, in a mouse model of Parkinson’s disease [25].

In summary, EVs have great potentialities as disease biomarkers as well as delivery tools for therapeutic/immunogenic molecules.

## 5. The EV-Based Vaccine Platform

**Figure 4:**
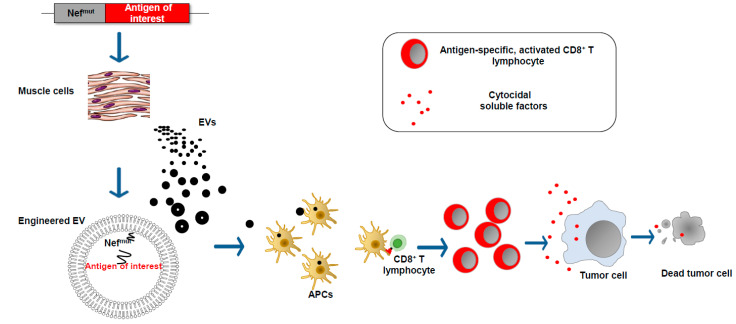
EV-based CD8^+^ T cell-specific vaccine platform. The intramuscular injection of a DNA vector leads to the expression of the antigen of interest fused at the C-terminus of the EV-anchoring protein Nef^mut^. EVs emerging from muscle cells incorporate the fusion product at high levels. The entry of engineered EVs into APCs leads to the cross-presentation of the antigen of interest with the activation of antigen-specific CD8^+^ T lymphocytes.

We found that an HIV-1 Nef mutant called Nef^mut^ incorporates into EVs at quite high levels, meanwhile losing most of the biologic functions of its wild-type counterpart. These characteristics are conserved when a heterologous protein is fused to its C-terminus [26]. Therefore, this 27 kilodalton protein mutant can be considered a powerful EV-anchoring protein. On this basis, we developed an original vaccine platform based on the intramuscular injection of a DNA vector coding for Nef^mut^ (Figure 4). Both N-terminal myristoylation and palmitoylation fasten Nef^mut^ to the luminal membrane leaflets and are critical for its abundant upload in EVs. Nanovesicles containing Nef^mut^-fused antigens released by muscle cells can freely circulate in the body and be internalized by APCs. EV-associated antigens can then be cross-presented to prime antigen-specific CD8^+^ T cells. Notably, a Nef^mut^ isotype with a 21-amino acid C-terminal truncation maintains both EV-anchoring and immunogenic properties, thus representing a safer alternative for use in clinical practice [27].

Early in vitro experiments strongly supported the idea that the entry of Nef^mut^-engineered EVs into APCs results in the cross-presentation of EV-incorporated antigens and the activation of antigen-specific CD8^+^ T lymphocytes [26]. CD8^+^ T cell immunogenicity was then confirmed in in vivo experiments, first by injecting in vitro-engineered EVs and afterward by engineering EVs spontaneously released by muscle cells upon the injection of DNA vectors expressing Nef^mut^-based fusion products (Figure 4) [28].

On the basis of both data from the literature and experimental evidence, a model for the mechanism of the cross-presentation of the antigens uploaded in Nef^mut^-engineered EVs can be envisioned. After their release from muscle cells, engineered EVs can enter APCs via endocytosis. EV cell internalization can be followed by degradation into late endosomes/lysosomes, ultimately leading to the delivery of peptides to the endoplasmic reticulum (ER) for complexing with MHC Class I molecules at the completion of the vacuolar cross-presentation pathway. Alternatively, the internalized EVs can undergo fusion with the membranes of endosomes, as described for several viruses. In this way, Nef^mut^-based fusion products are exposed to cytoplasm, thus becoming vulnerable to proteasome degradation. The resulting peptides can be then translocated to the ER by the transporter associated with antigen processing (TAP) for their complexing with MHC Class I molecules to initiate cross-priming events.

The Nef^mut^-based strategy was successfully applied against transplantable tumors expressing TSAs, i.e., HPV-E6 and -E7 [29], as well as against ectopic tumors expressing HER2/neu [30], i.e., a TAA overproduced in a model of a spontaneous mouse mammary tumor. Of note, recent investigations we carried out with viral antigens demonstrated that the Nef^mut^-based platform can be employed contemporarily against up to four antigens without any reciprocal negative interference in terms of immunogenicity [31]. In addition, the intramuscular quadricep injection of Nef^mut^-based DNA vectors generated a strong cell immune response in distal districts (i.e., the lungs) which are typically poorly infiltrated by circulatory immune cells [32]. We assume that this effect was a consequence of the penetration of immunogenic EVs into distal tissues, as previously described in biodistribution studies carried out with fluorescently labeled EVs injected intravenously [33,34,35,36,37,38,39]. Furthermore, the EV-induced immunization generated a long-lasting immune memory in the form of antigen-specific CD8^+^ T-resident memory lymphocytes. On this subject, we demonstrated that that the generation of endogenous EVs engineered for the incorporation of Severe Acute Respiratory Syndrome Coronavirus (SARS-CoV)-2 Nucleocapsid (N) protein induced long-lasting immunity in the lungs of K18-hACE2 transgenic mice which was associated with control over viral replication. The antiviral effect also remained effective when the viral challenge was carried out 3 months after boosting and when coupled with the persistence of N-specific CD8^+^ T-resident memory lymphocytes [32].

In what way can these characteristics be exploited to overcome the current limitations of anticancer immunotherapies?

First, by virtue of the possibility of inducing CD8^+^ T cell immunity against different antigens contemporarily, the Nef^mut^-based antitumor strategy would efficiently counteract the tumor cell escape phenomenon often occurring in immunotherapies targeting a single tumor marker. This characteristic would be applied also to target a quite high number of tumor neo-antigens whose sequences can be included in different expression vectors to be injected simultaneously.

In addition, different to what is often detected in humans with either ICI- or CAR-T cell-based treatments, our observations, made on more than one thousand small animals, excluded the idea that the generation of immunogenic EVs couples with systemic, unspecific immune activation.

Furthermore, Nef^mut^-based immunization is also able to generate antigen-specific resident memory CD8^+^ T cells (Trm lymphocytes, i.e., a CD8^+^ T cell sub-population co-expressing CD49a, CD69, and CD103 markers) in distal tissues [32]. This evidence supports the idea that repeated immunizations, as, for instance, practiced in the case of mRNA-based vaccines [40], would not be necessary to maintain antitumor protection over time. It is worth noting that the presence of tumor-specific CD8^+^ Trm cells in cancer patients is now considered a strong predictor of survival [41,42] since these cells play a major role in patrolling the growth of solid tumors, thus precluding the development of clinically relevant pathologies [43].

Finally, the quite low cost of both the production and storage of the immunogen, i.e., a simple, single-promoted DNA vector, certainly represents an additional advantage.

In summary, this EV-based vaccine platform offers several potential advantages over current cancer immunotherapies. Therefore, it deserves accurate clinical testing with the prospective of becoming a new weapon able to complement current antitumor approaches.

## 6. Still Unresolved Issues: An Option to Counteract Immunosuppression in the Tumor Microenvironment

The efficacy of cell-based anticancer immunotherapies is often restricted by the occurrence of tumor escape. This process can take place by means of MHC downregulation with the consequent loss of TCR recognition, as well as, concerning CAR-T cell-driven immunotherapy, through the selection of null mutant tumor cells. In the former case, however, the NK-induced cytotoxic effect against MHC-defective cells can, at least in part, control the growth of escaping tumors.

Antitumor cell-based immunotherapies against solid tumors can encounter an additional and essentially still unresolved issue, i.e., immunosuppression in the tumor microenvironment (TME), which often generates conditions hindering the functions of infiltrated tumor-specific cytotoxic T lymphocytes.

In solid tumors, cancer cells are embedded within a milieu composed of both cellular and non-cellular components that favors their proliferation. Fibroblasts, endothelial cells, and immune cells with immunosuppressive functions including neutrophils, myeloid-derived suppressor cells, and CD4^+^ Treg lymphocytes, as well as M2-like tumor-associated macrophages (TAMs), are part of the TME [44]. These latter cells can represent up to 50% of a tumor’s mass and show a typical anti-inflammatory phenotype. TAMs play a key role in immune evasion in the TME by secreting proteases, angiogenic factors, and pro-tumoral products [45] and are characterized by the expression of mannose receptor (MR, CD205) [46]. In this scenario, an attractive strategy to counteract the immunosuppressive TME can consist of re-programming M2 macrophages toward an M1 inflammatory phenotype, supporting the functions of antitumor-specific lymphocytes much better.

Interestingly enough, the surfaces of EVs contain high amounts of mannose, allowing them to bind and enter TAMs in the TME efficiently [47]. On the other hand, the nanovesicle-driven entry into APCs of Nef^mut^ (both alone and as part of fusion products) leads to cell activation with the induction of related markers, as well as the release of inflammatory factors, which is likely the consequence of the activation of several signal transduction molecules, including STAT-1, -2, and -3, NF-kB, JNK, ERK1/2, and MAPK. On this basis, one may envision that the entry of Nef^mut^-engineered EVs into M2 macrophages in the TME can lead to a shift towards an M1-like inflammatory phenotype, hence contributing to the generation of a behavior more favorable for antitumor CTL activity. Ongoing investigations would offer an experimental confirmation of such an attractive hypothesis.

## 7. Conclusions

Strengthening the cell immune response against tumor cells is the last frontier of antitumor immunotherapies. A number of immune-stimulating strategies have been already applied in clinical practice with success, but some issues still need to be addressed. Flexibility, simplicity, specificity, and low cost distinguish EV-based immunotherapy technology from the other technologies currently in use in clinical practice. Hence, this antitumor vaccine platform has the potential to emerge as a novel additional weapon against both hematologic and solid cancers.

## Data Availability

No new data are created.
